# Angiotensin Converting Enzyme (ACE)-Peptide Interactions: Inhibition Kinetics, In Silico Molecular Docking and Stability Study of Three Novel Peptides Generated from Palm Kernel Cake Proteins

**DOI:** 10.3390/biom9100569

**Published:** 2019-10-04

**Authors:** Mohammad Zarei, Najib Bin Zainal Abidin, Shehu Muhammad Auwal, Shyan Yea Chay, Zaibunnisa Abdul Haiyee, Adi Md Sikin, Nazamid Saari

**Affiliations:** 1 Department of Food Science and Technology, School of Industrial Technology, Faculty of Applied Sciences, Universiti Teknologi MARA, Shah Alam 40450, Selangor, Malaysia; 2 Department of Food Science, Faculty of Food Science and Technology, Universiti Putra Malaysia, Serdang 43400, Selangor, Malaysia; 3 Department of Biochemistry, Faculty of Basic Medical Sciences, Bayero University, Kano 700231, Nigeria

**Keywords:** peptide, kinetics, molecular docking, angiotensin converting enzyme inhibitory activity

## Abstract

Three novel peptide sequences identified from palm kernel cake (PKC) generated protein hydrolysate including YLLLK, WAFS and GVQEGAGHYALL were used for stability study against angiotensin-converting enzyme (ACE), ACE-inhibition kinetics and molecular docking studies. Results showed that the peptides were degraded at different cleavage degrees of 94%, 67% and 97% for YLLLK, WAFS and GVQEGAGHYALL, respectively, after 3 h of incubation with ACE. YLLLK was found to be the least stable (decreased ACE-inhibitory activity) compared to WAFS and GVQEGAGHYALL (increased ACE-inhibitory activity). YLLLK showed the lowest K_i_ (1.51 mM) in inhibition kinetics study when compared to WAFS and GVQEGAGHYALL with K_i_ of 2 mM and 3.18 mM, respectively. In addition, ACE revealed the lowest $K_{m}^{\mathrm{app}}$ and $V_{\max}^{\mathrm{app}}$ and higher catalytic efficiency (CE) in the presence of YLLLK at different concentrations, implying that the enzyme catalysis decreased and hence the inhibition mode increased. Furthermore, YLLLK showed the lowest docking score of −8.224 and seven interactions with tACE, while peptide GVQEGAGHYALL showed the higher docking score of −7.006 and five interactions with tACE.

## 1. Introduction

Bioactive peptides are protein-based compounds having amino acid residues in the range of 2 to 20 and possess numerous biological effects on human health. They are produced from different protein sources via a variety of production approaches such as the use of proteolytic enzymes, fermentation using different bacteria or fungi and digestion of food proteins after their consumption [1,2]. The most common biological roles of bioactive peptides which have been previously reported in numerous studies are antihypertensive, antioxidative, anti-thrombotic, anti-carcinogenic, immunomodulatory effects as well as anti-inflammatory and anti-aging activities [3,4,5,6,7,8]. 

Antihypertensive peptides are natural alternatives to synthetic angiotensin-converting enzyme (ACE) inhibitors, of which the latter would produce mild to severe adverse side effects such as dry cough, dizziness, headaches and fatigue. Although the ACE inhibitory activity of bio-peptides are lower than synthetic ACE inhibitors, they are safer with a reduced health risk, less costly and provide additional nutritional benefits as a source of essential amino acids [9,10].

Peptides inhibit the angiotensin-converting enzyme (ACE) in different ways. While some remain intact upon reaction with ACE, others are degraded by ACE and based on the structure, size and amino acid composition of the new peptides generated, their antihypertensive activity will be lower, higher or equal in comparison to their parent peptides. Hence, peptides are classified into three categories based on the alterations in their antihypertensive activity after hydrolysis by ACE, which include substrate type, true inhibitor type and pro-drug type [10,11].

Despite the available literature on different approaches for the production and isolation as well as on the bioavailability and bio-accessibility of food-derived bioactive peptides, there is still limited information and knowledge on the structure-function relationship, ACE inhibition kinetics study and the mechanism of actions of antihypertensive peptides. The structure-function relationships study gives information on how the amino acid composition of peptide sequences (as substrate) and ACE (as an enzyme) interact together and affect the overall ACE inhibitory activity. Additionally, the kinetics parameters such as K_m_, V_max_ and catalytic efficiency (CE) are calculated to determine the optimal peptide dosage which exhibits maximum ACE inhibitory activity [12,13]. Moreover, the mode of inhibition between ACE-peptide interactions is determined using Michaelis-Menten and Lineweaver-Burk plots obtained from kinetics studies.

Molecular docking study is performed mainly to investigate the binding mode between target-ligand interaction. Docking is a computer-based method that predicts the affinity and binding conformation of a small molecule ligand (an inhibitor) to the appropriate target binding site (an enzyme) in order to determine and evaluate the hydrogen bond, hydrophobic interaction, electrostatic interactions, Van der Waals interaction force and total energy between peptide and ACE [14,15]. Recently, in silico molecular docking has been used to study the interaction between inhibitory peptides and ACE in pistachio hydrolysate [15], *Salmo salar* [16], milk protein [17], hemp seed [12] and stone fish hydrolysates [18]. 

To our knowledge, there is no report on the inhibitory interaction between papain-generated bioactive peptides and ACE. Therefore, in the present study, interaction between ACE and three novel bioactive peptides of sequences YLLLK, WAFS and GVQEGAGHYALL, generated from palm kernel cake protein [19,20], is investigated. Peptides are then classified into substrate, prodrug and true inhibitor type, based on their stability against ACE. Inhibition kinetics study on the interaction between ACE and the peptides is also carried out to determine K_m_, V_max_, K_i_, and CE and mode of inhibition. Moreover, in silico molecular docking study for the interaction between ACE and each of the three bioactive peptides is performed.

## 2. Materials and Methods

### 2.1. Chemicals

Hydrochloric acid, pyridine, benzene sulfonyl chloride (BSC) and trifluoroacetic acid (TFA) were purchased from Fisher Scientific (Atlanta, GA, USA). Angiotensin-I-converting enzyme (rabbit lung), captopril and Hippuryl-l-Histidyl-l-Leucine (HHL) were obtained from Sigma Chemical Co., (St Louis, MO, USA). Sodium chloride, boric acid and sodium borate were purchased from Merck Co. (Darmstadt, Hesse, Germany). Peptide sequences YLLLK, WAFS and GVQEGAGHYALL were identified in the papain-generated protein hydrolysate from palm kernel cake (PKC) after fractionation and then were synthesized (First Base Laboratories Sdn Bhd, Selangor, Malaysia) to be used in this study.

### 2.2. Peptide Stability Study

Peptide stability study was carried out using the method described by Forghani et al. [21] and Yang, Marczak, Yokoo, Usui and Yoshikawa [22] with modifications. Each peptide solution (225 µL), at a concentration of 4 mM, was incubated with 150 µl of ACE (100 mU/mL) at 37 °C and incubation time of 0, 0.5, 1, 2 and 3 h. Peptide samples were withdrawn at their respective incubation time separately and kept in ice prior to analysis by HPLC. A 20-µL sample was loaded onto a Hypersil GOLD C18 column (4.6 × 250 mm) attached to a LC 20AT apparatus (Shimadzu Co., Kyoto, Japan). The column was conditioned with eluent A (0.1% TFA in DW) and eluted with 100% eluent A from 0 to 10 min. The peptides were eluted with eluent B (0.1% TFA in CH_3_CN) with a gradient elution of 0–100% for 10–50 min. The absorbance was read at 215 nm. 

### 2.3. Kinetics Study of ACE Inhibition

Mode of enzyme inhibition, maximum initial velocity (V_max_), Michaelis-Menten constant (K_m_), inhibitory constant (Ki) and catalytic efficiency were determined using Michaeles-Menten and Lineweaver-Burk plots of 1/v versus 1/[S]. ACE inhibition of peptides YLLLK, WAFS and GVQEGAGHYALL was determined in the presence of five concentrations of HHL including 0.5, 1, 2 and 8 mM and four concentrations of peptides in the range of 31 to 2000 µM (Table 1).

Enzyme activity was shown as nmol hippuric acid produced per min of enzymatic reaction. Michaelis-Menten and Lineweaver-Burk plots were created using GRAPHPAD PRISM 6.07 (GraphPad, Software Inc., San Diego, CA, USA) software. Vmax and Km were calculated using the same software. K_iu_ was calculated from the plot of 1/Vmax against inhibitor concentration at the intercept on the inhibitor concentration axis [23]).

### 2.4. Molecular Docking Studies

Molecular docking was conducted using Glide (Glide, version 6.7, Schrödinger, LLC, New York, NY, 2015). The structures of YLLLK, WAFS and GVQEGAGHYALL were generated using Molecular Operating Environment software (MOE 2014.0901, Chemical Computing Group, Inc., Montreal, QC, Canada, 2014), and the energies were minimized using MMFF94 program. For ACE docking, a crystal structure of human ACE bound to captopril as the ACE-inhibitory drug (PDB ID 1UZF) was employed as an ACE model molecule. A binding site was generated by digitally removing captopril within a radius of 20 Å, with the coordinates *x* = 36.98, *y* = 27.05, and *z* = 50.65. Glide extra precision (XP) docking was performed for all the ACE inhibitory peptides using captopril as a benchmark. All water molecules in the structure of protein were digitally detached, and hydrogen atoms were added to the structure of the protein. Binding energy values and the scores were used to evaluate the molecular docking to determine the best poses for each of the peptides. The binding affinity between the ACE and peptides were determined and reported as negative values of Glide scores (kcal/mol). The Glide scores (kcal/mol) are shown as negative values, and those positions with a more negative value exhibit a potent interaction between protein and ligand. The Glide pose viewer was employed to analyze the resulting docked poses and to detect the hydrogen bonds and the hydrophobic, hydrophilic, electrostatic, and coordination interactions between the ACE and peptides. The best docking poses with low Glide scores and the least binding energy were subjected to further analysis.

### 2.5. Statistical Analysis

The statistical analysis was carried out using Minitab 16.0 software (MINITAB, State College, PA, USA). All experiments were performed in triplicate. Data are presented as mean ± standard deviation from at least triplicate determinations. Analysis was carried out using one-way analysis of variance (ANOVA). Statistically significant differences among means of experimental design were studied by Tukey multiple range tests at *p* = 0.05 using MINITAB RELEASE 16.

## 3. Results and Discussion

### 3.1. Effect of the Catalytic Activity of Angiotensin Converting Enzyme on Bioactive Peptides

**Figure 1:**
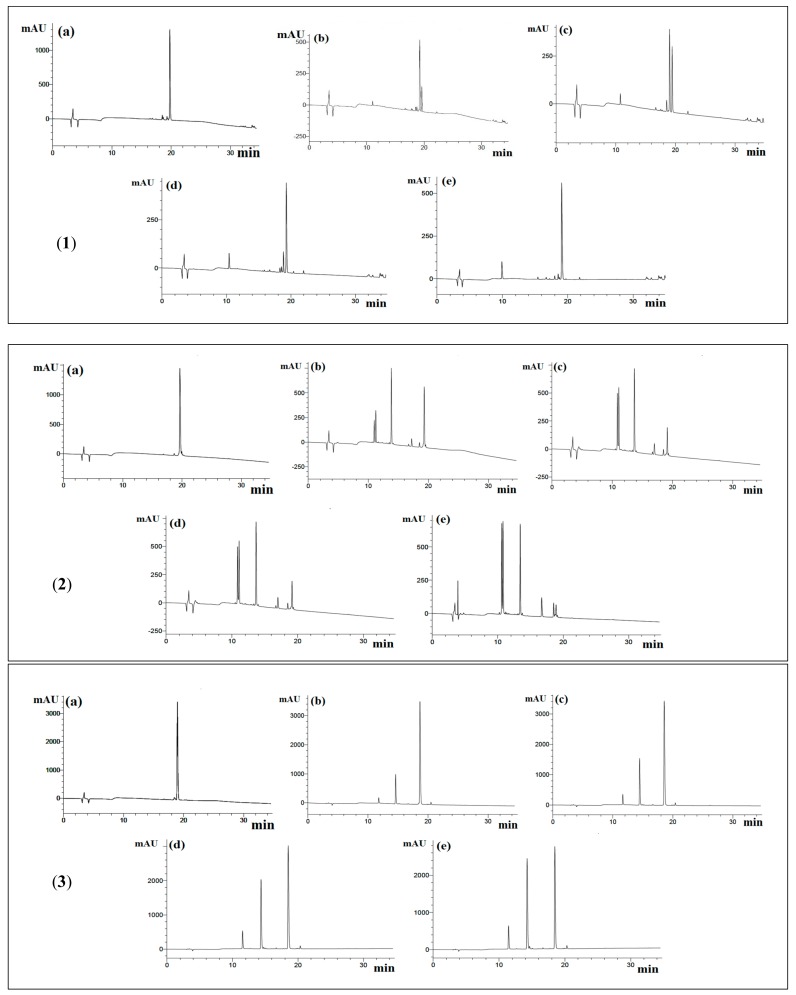
**Reversed-phase high performance liquid chromatography** (RP-HPLC) chromatograms of hydrolysis of peptides by ACE. (**1**) YLLLK; (**2**) WAFS (**3**) GVQEGAGHYALL; (**a**) before incubation; (**b**) 0.5 h of incubation; (**c**) 1 h of incubation; (**d**) 2 h of incubation; (**e**) 3 h of incubation. 20 μL of sample was eluted by mobile phase A (0.1% of TFA in deionized water) and 0–60% gradient of mobile phase B (0.1% of TFA in CH_3_CN) for 5–35 min on a C18 column.

From HPLC chromatograms, YLLLK showed that the number of peaks was 1 at the first 30 min with 91.7% of peptide cleavage and finally it decreased to 4 peaks in 3 h of pre-incubation with 94.3% cleavage, while WAFS and GVQEGAGHYALL showed 3 and 6 peaks, respectively, throughout 3 h of incubation. Furthermore, the percentage cleavage for WAFS and GVQEGAGHYALL was 43% and 46% in the first 30 min of incubation time and 68% and 97%, respectively after 3 h of incubation time, implying that none of these peptides is resistant to ACE, thus eliminating the possibility of “true inhibitor” (Figure 1 and Table 2).

In a study performed by Skidgel and Erdös [24], they proposed that Substance P, a neuropeptide with the sequence of RPKPQQFFGLM, can be cleaved by ACE from the penultimate residue with the removal of LM from the sequence since ACE is a peptidyl dipeptidase that removes C-terminal dipeptides from its substrates, such as bradykinin and angiotensin I [25,26]. Therefore, it degraded peptide RPKPQQFFGLM into two fractions of RPKPQQFFG and LM. Similarly, YLLLK also has been degraded by ACE into YLL and LK. Moreover, in another research, Rao, et al. [27] studied the effect of ACE on a synthesized peptide, IKPFR, and they observed that it was degraded into two fragments of IKP and FR. It seems that peptide WAFS also was degraded into two sequences of WA and FS due to having a phenylalanine (F) residue in the second position of the peptide sequence from its C-terminus. It was shown that bradykinin, which contains a phenylalanine residue at the second position from C-terminus, was broken into two fragments of RPPGFSP and FR [28], which supported the observation in the current study.

It seems that peptide GVQEGAGHYALL has been degraded by ACE at different positions. Similar results have been obtained from incubation of ACE with different substrates such as Substance P [24], RMLGQTPTK [29] and FKGRYYP [30], suggesting that ACE can degrade the sequences containing the amino acid residues L, Y and G in their structure. 

Peptide YLLLK showed 100% ACE inhibitory activity before pre-incubation whereas the ACE inhibitory activity dropped to 80% after pre-incubation with ACE. Peptides WAFS and GVQEGAGHYALL showed higher ACE inhibitory activity after pre-incubation, suggesting that peptide YLLLK as a substrate type inhibitor whereas peptides WAFS and GVQEGAGHYALL are pro-drug type inhibitors due to higher ACE inhibitory activity after pre-incubation (Table 3).

### 3.2. Inhibition Kinetics of Bioactive Peptides toward ACE

Lineweaver-Burk and Michaelis-Menten plots were used to determine the inhibition kinetics of YLLLK, WAFS and GVQEGAGHYALL. The calculated K_m_ (Michaelis constant), V_max_ (maximum reaction velocity), CE (catalytic efficiency) and K_iu_ (enzyme-inhibitor dissociation constant) values for the bioactive peptides are summarized in Table 4.

The K_m_ value for angiotensin-converting enzyme activity in the absence of inhibiting peptides YLLLK, WAFS and GVQEGAGHYALL was around 1.458, while in the presence of these peptides at the concentrations of 31 to 250 µM, 62 to 2000 µM and 31 to 250 µM, $K_{m}^{\mathrm{app}}$ was calculated between 0.3257 to 1.7820 mM, 2.807 to 5.712 mM and 1.890 to 2.856 mM, respectively. As shown, the K_m_ value for ACE activity alone was lower than $K_{m}^{\mathrm{app}}$ for ACE in the presence of peptides, indicating that the ACE reaction requires more substrate for catalysis in the presence of peptides WAFS and GVQEGAGHYALL at all concentrations and YLLLK only at concentration of 31 µM. The $K_{m}^{\mathrm{app}}$ value for concentration of 62, 125 and 250 µM in the presence of peptide YLLLK was lower than the K_m_ value of ACE in the absence of YLLLK. 

The $K_{m}^{\mathrm{app}}$ value reduced when concentration of peptides YLLLK and GVQEGAGHYALL increased, implying that at higher peptide levels, more peptides become bound to the enzyme active site to prevent the formation of an enzyme-substrate complex. 

The V_max_ for uninhibited ACE was 31 nmol/min. In the presence of three peptides, V_max_ decreased when peptide concentrations increased. Therefore, the results revealed that the activation energy was increased in the presence of the peptides because the velocity of the enzyme reaction was decelerated. This result is in agreement with that reported by Girgih, He and Aluko [12] and Forghani, Zarei, Ebrahimpour, Philip, Bakar, Abdul Hamid and Saari [21]. In particular, peptide YLLLK showed lower $V_{\max}^{\mathrm{app}}$ in comparison to the other two peptides at all concentrations, which is in agreement with its lower IC_50_ (47 µM) and higher ACE inhibitory activity.

**Figure 2:**
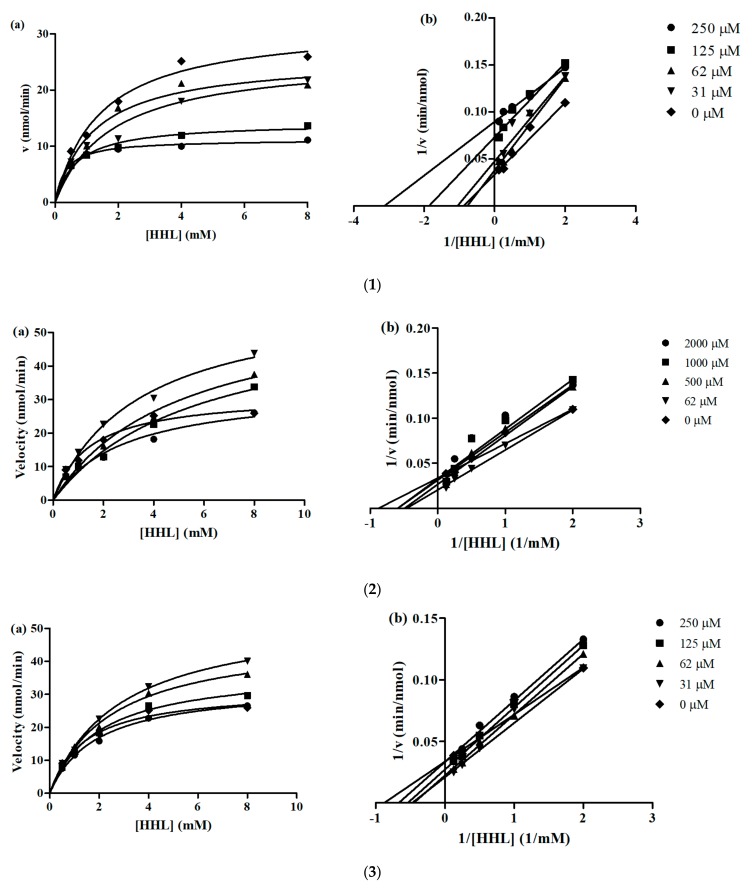
(**a**) Michaelis-Menten and (**b**) Lineweaver-Burk plot of peptides. Each point represents the mean of three experiments. ACE activities measured in the absence or presence of peptides. (**1**) YLLLK; (**2**) WAFS and (**3**) GVQEGAGHYALL.

Furthermore, kinetic studies showed that the inhibition mode of YLLLK, WAFS and GVQEGAGHYALL, at different concentrations, was mixed-type inhibition because the *x*-axis and slope lines of Lineweaver-Burk plots do not intersect at the same point and the K_m_ values of the control and various peptide concentrations were different (Figure 2). This demonstrates that the peptides can bind both to the free enzyme as well as to the ES complex.

The catalytic efficiency (CE) of uninhibited ACE reaction for YLLLK, WAFS and GVQEGAGHYALL was 21.81, 21.88 and 22.69, respectively. These peptides demonstrated lower CE values in all peptide concentrations compared to uninhibited ACE, except concentration of 250 µM for YLLLK, indicating that enzyme catalysis was lower in the presence of peptides compared to that in the absence of peptides. Thus, the peptides demonstrated some binding affinity to the enzyme, which led to reduced catalytic ability and consequently reduced their $V_{\max}^{\mathrm{app}}$ and IC_50_ values. Peptide YLLLK showed the lowest K_iu_ (0.155 mM) compared to the peptides WAFS and GVQEGAGHYALL with K_iu_ 2 mM and 3.18 mM, respectively. The lower K_iu_ value for YLLLK is consistent with the observed greater reductions in V_max_ and CE when compared to the reductions produced by WAFS and GVQEGAGHYALL. The K_iu_ value obtained for peptide YLLLK (0.155 mM) was lower than the K_iu_ value of CRQNTLGHNTQTSIAQ (0.21 mM), VSRHFASYAN (0.61 mM), SAAVGSP (1.57 mM) generated from *Stichopus horrens* [21] and TF (12.2726 mM). However, it was higher than WVYY (0.06 mM), which was previously reported by Udenigwe, et al. [31]. Peptide GVQEGAGHYALL showed the highest K_iu_ (3.18 mM) compared to peptides YLLLK and WAFS, indicating that this peptide has the lowest affinity towards ACE in this study. This peptide also showed the highest IC_50_ (239 µM) among three peptides.

### 3.3. Molecular Docking Studies

**Figure 3:**
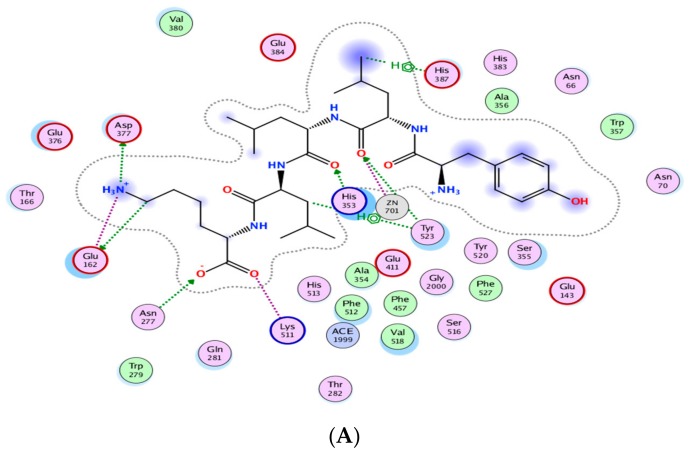

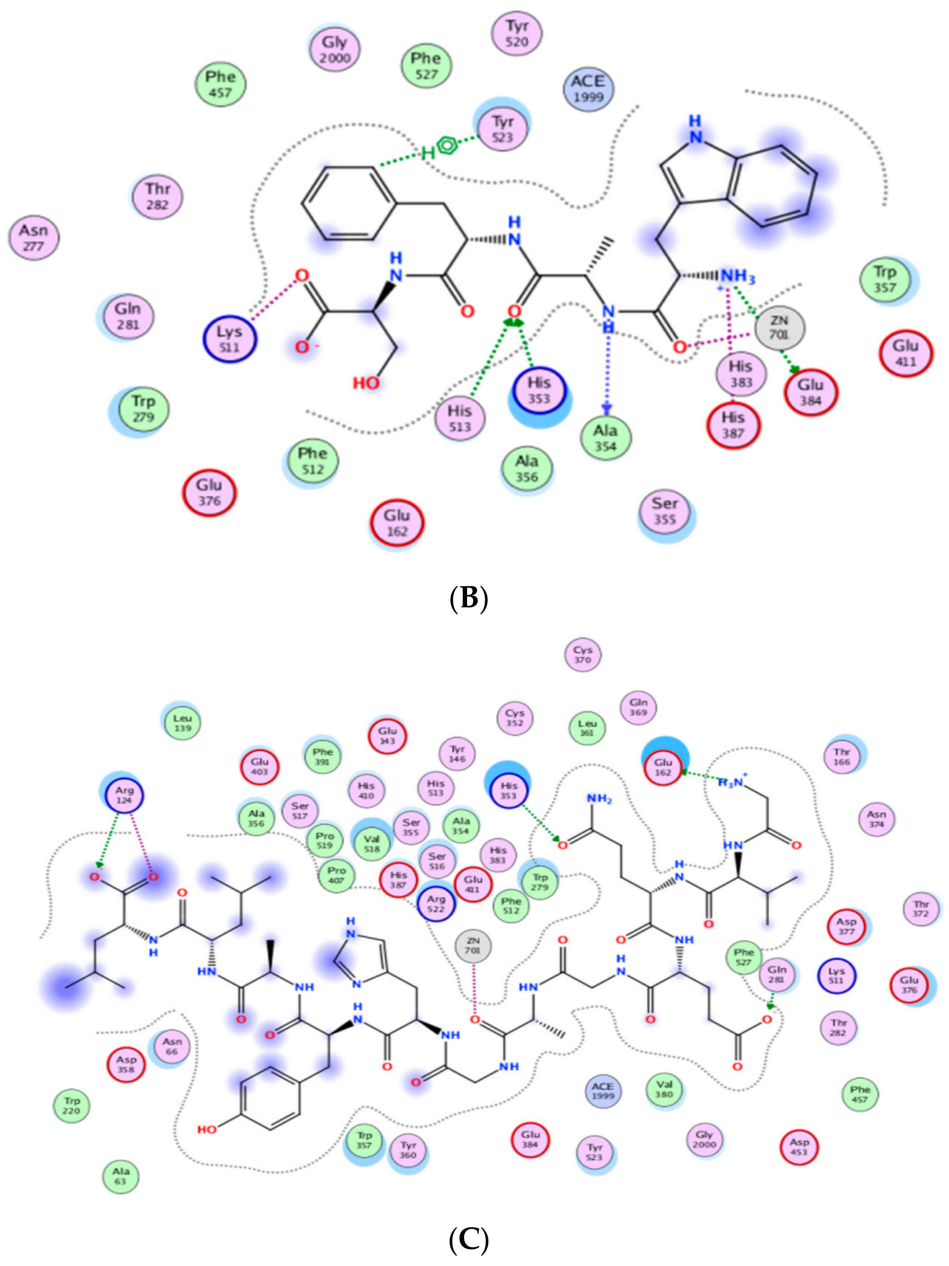
Automated molecular docking of YLLLK (**A**), WAFS (**B**), and GVQEGAGHYALL (**C**) at the angiotensin-converting enzyme (ACE) active site. ACE hydrophobic residues are represented in green, positively charged residues in blue, and negatively charged residues in red; hydrogen bonds are purple arrows, polar residues are in turquoise color, and other residues and the zinc atom are represented automatically. Image obtained with Accelrys DS Visualizer software.

Figure 3 indicates the presence of the best poses for YLLLK, WAFS and GVQEGAGHYALL within the catalytic site of ACE. The stability of the best pose for each peptide was achieved through hydrogen bonding, electrostatic and hydrophobic interactions between amino acid residues of the ACE binding domain and those of the individual peptide within a distance of 3.5 Å. YLLLK exhibited the higher number of total interactions (7) with ACE, which could be the main reason for the higher ACE inhibitory activity (lower IC_50_ value of 47 µM) compared to WAFS and GVQEGAGHYALL, which have five interactions each. This was ascertained by the lowest electrostatic binding energy of −122.247 kJ/mol for YLLLK compared to −115.733 kJ/mol for WAFS and −115.0981 kJ/mol for GVQEGAGHYALL, as shown in Table 5. Similarly, the van der Waals energy of −4.115 kJ/mol for YLLLK was lower compared to WAFS (−2.267 kj/mol) and GVQEGAGHYALL (−1.396 kJ/mol). As shown in Table 4, the binding energy data revealed the higher ACE-binding affinity (lower K_i_ value of 0.155 mM) of YLLLK compared to WAFS and GVQEGAGHYALL with 2 and 3.18 mM, respectively. The higher activity of YLLLK may also be due to a higher degree of interactions with the active site of ACE. As shown in Table 5, it appears that the ability of peptides to develop numerous hydrogen bond interactions with ACE may be a major factor in the ACE inhibitory activity of the peptide and stabilization of the ACE-peptide complex structure. 

A similar pattern of hydrogen bonds with ACE residues has been reported for peptides WVYY (7 bonds) and WYT (5 bonds) [12]. Furthermore, the IC_50_ is correlated to the number of hydrogen bonds formed. As shown in Table 5, YLLLK revealed the lowest IC_50_ of 47 µM and formed 7 H-bonds, followed by WAFS, with an IC_50_ of 202 µM and GVQEGAGHYALL with 239 µM. Thus, it appears that the number of hydrogen bonds and ACE residues involved play a prominent role in the ACE-inhibitory capacity of the peptides.

## 4. Conclusions

Interaction of ACE with three novel papain-generated bioactive peptides from palm kernel cake proteins, namely YLLLK, WAFS and GVQEGAGHYALL, was studied by molecular docking simulation. Furthermore, the inhibition kinetics and the stability of the peptides against ACE were studied. Lineweaver-Burk plots revealed that the inhibition mode of the peptides at different concentrations was mixed-type inhibition, implying that the peptides can compete with the substrate to bind to the active site of the enzyme and at other non-active sites. Moreover, peptide stability study showed that the peptides were degraded to different cleavage degrees upon pre-incubation with ACE. Additionally, results demonstrated that YLLLK was a substrate inhibitor, whereas peptides WAFS and GVQEGAGHYALL were prodrug inhibitors. Based on molecular docking study, YLLLK exhibited a higher number of total interactions in comparison to WAFS and GVQEGAGHYALL, which could be a reason for the lower IC_50_ of YLLLK. 

## Figures and Tables

**Table 1:** Peptide concentrations used for kinetic study.

	Peptides	Peptide Concentration (µM)			
1	YLLLK	250.0	125.0	62.0	31.0
2	WAFS	2000	1000	500	62.0
3	GVQEGAGHYALL	250.0	125.0	62.0	31.0

**Table 2:** Hydrolysis of papain-generated peptides by angiotensin-converting enzyme (ACE) with incubation for 3 h.

Bioactive Peptides						
Incubation Time (h)	YLLLK		WAFS		GVQEGAGHYALL	
	Peaks	Cleavage (%)	Peaks	Cleavage (%)	Peaks	Cleavage (%)
0.5	2	92 ± 2.8	3	43 ± 2.0	6	46 ± 1.5
1	4	93 ± 1.9	3	42 ± 1.6	6	65 ± 2.6
2	3	94 ± 3.0	3	63 ± 3.0	6	86 ± 3.1
3	2	94 ± 2.7	3	68 ± 3.4	6	97 ± 3.4

**Table 3:** ACE-inhibitory capacities (%) of peptides with and without pre-incubation with ACE.

Peptides	ACE-Inhibitory Capacity (%)		Classification
	Without Pre-Incubation	With Pre-Incubation	
YLLLK	100 ± 2.99	80 ± 2.04	Substrate type
WAFS	55 ± 1.81	56 ± 1.34	Pro-drug inhibitor
GVQEGAGHYALL	70 ± 1.15	75 ± 2.11	Pro-drug inhibitor

**Table 4:** *V*_max_, *K*_m_ of ACE inhibited by peptides along with its *K*_iu_ and CE.

Peptide Sequence	Peptide Concentration (µM)	*K*_m_ (mM)	*V*_max_ (nmol/min)	$K_{m}^{\mathrm{app}}$ (mM)	$V_{\max}^{\mathrm{app}}$ (nml/min)	*CE* ($V_{\max}^{\mathrm{app}}$/$K_{m}^{\mathrm{app}}$)	*K*_iu_ (mM)
YLLLK	250 125 62.0 31.0			0.3257 0.6862 1.2430 1.7820	11.19 14.21 25.72 25.88	34.35 20.70 20.69 14.52	0.155
Control	0.00	1.452	31.67			21.81	
WAFS	2000 1000 500 62.0			2.807 5.712 5.051 3.455	33.63 56.81 59.79 61.03	11.98 9.94 11.83 17.66	2
Control	0.00	1.456	31.87			21.88	
GVQEGAGHYALL	250 125 62.0 31.0			1.890 1.973 2.483 2.856	32.83 37.77 47.69 54.68	17.37 19.14 19.20 19.14	3.18
Control	0.00	1.493	32.39			21.69	

**Table 5:** ACE inhibitory activities and docking study characteristics of peptides.

	YLLLK	WAFS	GVQEGAGHYALL
ACE Inhibition (%)	100	55	70
ACE Inhibition (IC_50_)	47	202	239
Electrostatic interaction (kJ/mol)	−122.247	−115.733	−115.098
Hydrophobic interaction (kJ/mol)	−1.048	−0.535	−0.512
Van der Waals (kJ/mol)	−4.115	−2.267	−1.396
Docking Score	−8.224	−7.611	−7.600
H-bonds	7	5	5
Total Interaction	15	15	13
